# Supplementary material for: CENPE Inhibition Leads to Mitotic Catastrophe and DNA Damage in Medulloblastoma Cells
Source: Cancers (Basel). 2021 Mar 1;13(5):1028. doi: 10.3390/cancers13051028 (PMC7957796; doi:10.3390/cancers13051028)
Supplement: Supplementary file 1 [file cancers-13-01028-s001.zip › cancers-1097588-supplementar material/cancers-1097588-final-suppl.docx]

Supplementary Material: CENPE Inhibition Leads to Mitotic Catastrophe and DNA Damage in Medulloblastoma Cells

Giorgia Iegiani, Marta Gai, Ferdinando Di Cunto and Gianmarco Pallavicini

**Figure S1.** The original western blot images. (**A**) The original image of Figure 3B (red squares), 6F (green squares) (**B**) The original image of Figure 7A (red squares), 7C (green squares) (**C**) The original image of Figure 7E (red squares) (**D**) The original image of Figure 7G (green squares).


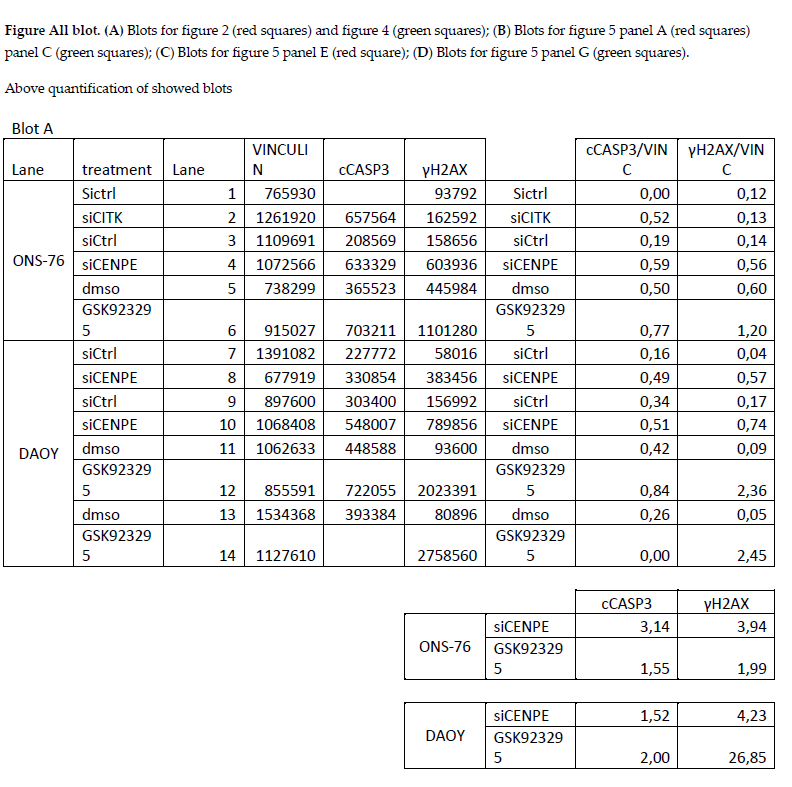


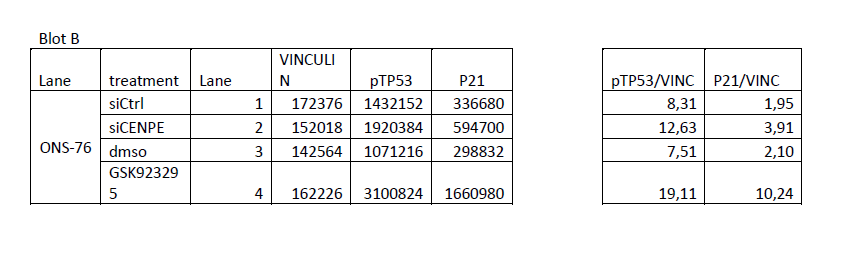


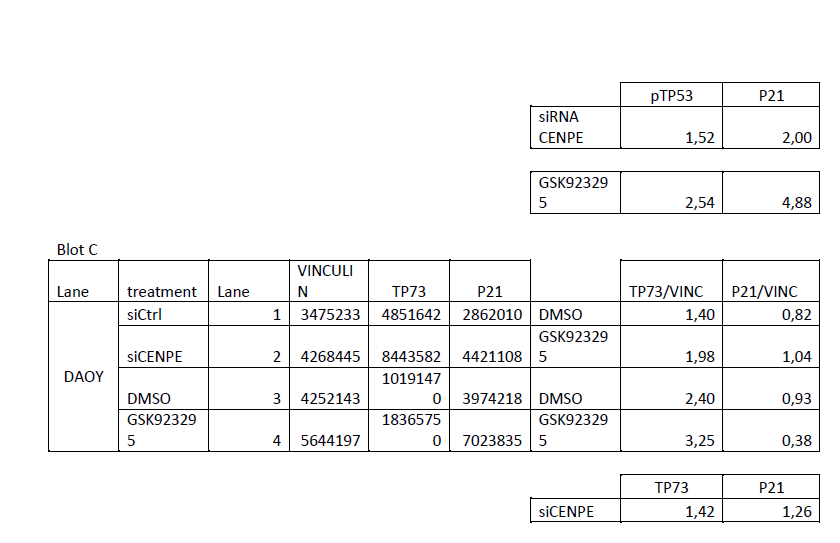


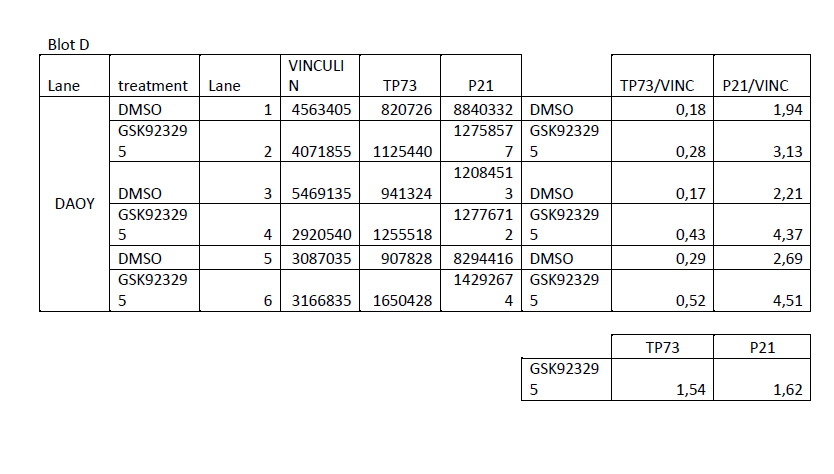


**Figure S2.** Quantification of all blots. (**Blot A**) Quantification for figure 3 panel B and figure 6 panel F; (**Blot B**) Quantification for figure 7 panel A and panel C; (**Blot C**) Quantification for figure 7 panel E; (**Blot** **D**) Quantification for figure 7 panel G.
